# Oxidative Stress and Its Implications in the Right Ventricular Remodeling Secondary to Pulmonary Hypertension

**DOI:** 10.3389/fphys.2019.01233

**Published:** 2019-09-24

**Authors:** Matthew Mikhael, Christian Makar, Amir Wissa, Trixie Le, Mansoureh Eghbali, Soban Umar

**Affiliations:** Division of Molecular Medicine, Department of Anesthesiology and Perioperative Medicine, David Geffen School of Medicine, UCLA, Los Angeles, CA, United States

**Keywords:** oxidative stress, right ventricle, pulmonary hypertension, remodeling, RV failure

## Abstract

Pulmonary hypertension (PH) is a pulmonary vascular disease characterized by increased pulmonary artery pressures. Long standing pulmonary arterial pressure overload leads to right ventricular (RV) hypertrophy, RV failure, and death. RV failure is a major determinant of survival in PH. Oxidative stress has been associated with the development of RV failure secondary to PH. Here we summarize the structural and functional changes in the RV in response to sustained pulmonary arterial pressure overload. Furthermore, we review the pre-clinical and clinical studies highlighting the association of oxidative stress with pulmonary vasculature and RV remodeling in chronic PH. Targeting oxidative stress promises to be an effective therapeutic strategy for the treatment of RV failure.

## Pulmonary Hypertension and Its Effects on the Right Ventricle

Pulmonary hypertension (PH) is characterized by an average mean pulmonary artery pressure of 25 mmHg or greater at rest. Long standing right ventricular (RV) pressure overload in PH leads to RV hypertrophy and eventually RV failure and death (Grosse et al., 2017). Early detection of PH can lead to a better prognosis and increase in the average survival rate (McLaughlin, 2013).

**FIGURE 1:**
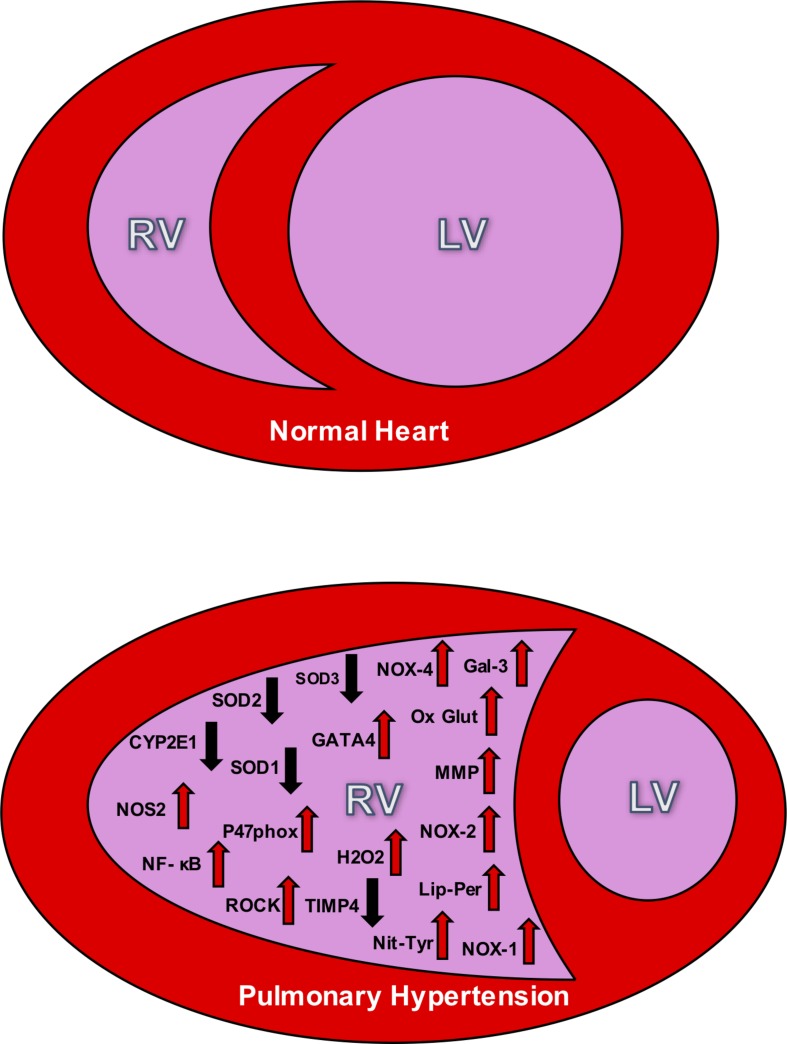
The **top** panel depicts the relative size difference between the right ventricle (RV) and the left ventricle (LV) in a normal heart. The **bottom** panel shows the effect of RV hypertrophy and the change in structure that accompanies PH. This **bottom** figure also illustrates the oxidative stress markers seen in the RV during PH. The up arrows signify increases in the markers during PH, and down arrows signify decreases. NOX, NADPH oxidase; SOD, superoxide dismutase; NOS, nitric oxide synthase; Ox Glut, oxidized glutathiones; TIMP, tissue inhibitor of metalloproteinase; MMP, matrix metalloproteinase; ROCK, Rho kinase; H_2_O_2_, peroxide; Lip-Per, lipid peroxidation; Nit-Tyr, nitrotyrosine. Sources: Gal 3 (He), NOX-4 (He, Cowley, Li), SOD3 (Zelko), SOD2 (Maron), SOD 1 (Cowley), GATA4 (Park and Suzuki), CYP2El (Potus), NOS2 (Dunlop), NF- κB (Boehm), oxidized glutathiones (Wang, Türck), nitrotyrosine (Qipshidze, Demarco), TIMP4 (Qipshidze), MMP (Qipshidze), NOX-2 (Ikeda,Li), p47phox (Ikeda), Rho kinase (Ikeda, Alzoubi), NOX-1 (Galhotra, Meghwani, Türck), hydrogen peroxide (Brandes), and lipid peroxidation (Puukila).

Considering healthy pulmonary circulation maintains low pressure compared to the systemic circulation, the RV walls are thinner and less resilient than the left ventricular (LV) walls (Badano et al., 2010). In response to chronic pressure overload due to PH, the RV undergoes adaptations including myocardial hypertrophy, followed by contractile impairment, and activation of myocardial renin-angiotensin-aldosterone system. Despite all these adaptations, RV may still exhibit dysfunction due to afterload mismatch. This RV dysfunction can be attributed to a reduced cardiac output, and functional tricuspid regurgitation. As hypertrophy and dilation continue to affect the RV, the RV becomes more spherical and its cross-sectional area enlarges, and the interventricular septum flattens; ultimately causing LV dysfunction (Badano et al., 2010; Figure 1).

## Pulmonary Hypertension-Induced Right Ventricular Hypertrophy

Right ventricular hypertrophy (RVH) is an adaptive transformation in the RV tissue that is usually triggered by chronic pressure overload. This pressure overload can result from stenotic valvular heart diseases or more commonly, pulmonary vascular disease associated with PH (Ikeda et al., 2014; Avazmohammadi et al., 2017). Pressure overload induces different responses in the LV and the RV due to their differing embryonic formations. Both chambers typically start developing at a 3.5 mm thickness prenatally but will grow at different rates after birth. The RV will grow to a 4 mm thickness while the LV quadruples in size to about 11 mm (Ryan and Archer, 2014). Thus in order to accommodate for the higher chronic pulmonary arterial pressures that accompany the different types of PH, these two relative thicknesses will change.

Right ventricular cardiac output depends on the synchronization between the force of contractile myocytes and the resistance to the blood flow through the pulmonary system. Normally, the RV can accommodate large but acute changes in volume loading during high levels of physical exercise, for example. However, there is a limited contractile reserve to keep the ejection fraction at homeostatic levels. In clinical cases and pre-clinical studies of animal models, the dilation of the RV is proportional to the severity of the adjacent vascular pressures (Watts et al., 2010). Hence, as afterload increases, the RV must significantly increase in size in order to maintain a suitable stroke volume. As a result, the naturally low pressure and elastic RV becomes large and inelastic, pushing on the interventricular septum to subsequently decrease the size of the LV (see Figure 1).

Some patients can live for years with adaptive RV hypertrophy by overcoming the high pressures, but when the RV becomes maladaptive, the complications follow soon afterward. Despite numerous studies highlighting the structural and functional changes of the RV secondary to PH, the transcriptomic profile, and molecular pathways that regulate these changes in the RV are not well studied (Lahm et al., 2018). For example, regulators such as extracellular ubiquitin, STAT3, Nrf2, and Ang-2/Tie-2 have all been identified as angiogenic regulators that contribute to LV morphological change, but it is not known whether these same pathways are regulating the RV as well (Lahm et al., 2018).

## Right Ventricular Failure Secondary to Chronic Pulmonary Hypertension

Right ventricular function is the leading prognostic determinant of survival in PH patients. For example, 5-year survival in patients with pulmonary arterial hypertension (PAH) is strongly correlated with RV ejection fraction (RVEF) compared to pulmonary artery pressures or pulmonary vascular resistance (van de Veerdonk et al., 2011). However, there is no true definition for right ventricular failure (RVF) as it clinically varies from case to case and the therapeutic options for RVF are very limited. Recently, the American Thoracic Society defined RVF as “*a complex clinical syndrome characterized by insufficient delivery of blood from the RV in the setting of elevated systemic venous pressure at rest or exercise.*” This widespread phenomenon (affecting nearly 70 million in the United States alone) is a direct result of RV hypertrophy as the overworked myocytes experience increased afterload and lose their contractility (van de Veerdonk et al., 2011). While RV hypertrophy is just the initial compensatory mechanism for high pulmonary vascular pressures, RVF takes effect once the hypertrophy becomes maladaptive.

## Role of Oxidative Stress in the Lungs and Pulmonary Vasculature in Pulmonary Hypertension

Oxidative stress occurs when the number of reactive oxygen species (ROS) exceeds that which the biological system can combat and neutralize. ROS are oxygen containing molecules with an odd number of electrons, termed free radicals. These free radicals can oxidize molecules, such as lipids and DNA, causing a multitude of harmful effects. The pulmonary vasculature undergoes morphological changes in PH that can be mediated by oxidative stress. Past research has shown the expression of various oxidative stress markers changes in the lungs and pulmonary vasculature of animals and humans with PH.

In animal studies, Xu et al. (2011) found that increased oxidative stress contributes to the PH development in rodents. In normal conditions, extracellular superoxide dismutase (SOD3) is expressed in high concentrations in the lungs and is responsible for removing extracellular superoxide anions, a type of free radical contributing to oxidative stress. The absence of SOD3 resulted in significantly worse PH in hypoxic SOD3 knockout mice and MCT rats with a SOD3 loss-of-function gene mutation (Xu et al., 2011). In another study, Wang L. et al. (2017) examined the effects of 17β-estradiol and 2-methoxyestradiol on the oxidative stress-hypoxia inducible factor-1 (OS-HIF-1) pathway in rats with hypoxia-induced PH. Hypoxic rats had a significant increase in oxidative stress levels as indicated by increased serum ROS levels, decreased serum SOD, and decreased manganese SOD (MnSOD) levels. Furthermore, MnSOD mRNA and protein levels were decreased in the lung tissue (Wang L. et al., 2017).

Bertoli et al. (2018) investigated the effects of chronic iron-overload on the resistance pulmonary arteries in rats. They found that rats treated with a high dose of iron dextran demonstrated increased vasoconstriction and vascular hyper-reactivity and reduced NO which were reversed by antioxidant therapy (Bertoli et al., 2018). Hoshikawa et al. (2001) found that the free radical phosphatidylcholine hydroperoxide (PCOOH) increased within 7 days following hypoxic exposure in rat lungs, and treating the rats with antioxidant N-acetylcysteine resulted in suppression of lung PCOOH levels. The activity of xanthine oxidase (XO), an enzyme that generates ROS, was also increased in rat lung tissue over time from day 1 through day 21 of hypoxia. Treating rats with the XO inhibitor allopurinol significantly inhibited the hypoxia-induced PH and resulted in decreased lung PCOOH levels (Hoshikawa et al., 2001).

Shi et al. (2018) used Baicalein, a flavonoid with known anti-proliferative and anti-inflammatory effects, to reduce oxidative stress in the lungs in an attempt to ameliorate hemodynamic and pulmonary vascular changes associated with MCT-induced PH in rats. Baicalein treatment inhibited inflammatory biomarkers (such as IL-6, TNF-α, and IL-1β) and reduced the Bax/Bcl-2 ratio and levels of cleaved caspase-3 which led to an increase in SOD production resulting in lowered ROS and inhibition of the NF-κB pathway (Shi et al., 2018).

On review of human studies, Bowers et al. (2004) demonstrated that histological lung sections from patients with severe PH exhibited increased oxidative stress than healthy controls, and chronic prostacyclin infusion, a commonly prescribed therapy for PH, exerted an anti-inflammatory effect.

Preterm infants with persistent pulmonary hypertension of the newborn (PPHN) present distinct challenges due to their lack of antioxidant defense mechanisms. Wedgwood et al. (2019) argued that oxidant stress in PPHN results from free radical generation from underlying lung disease or from free radicals generated by supplemental oxygen. These free radicals in turn act on the NO pathway resulting in decreased cGMP levels and pulmonary vasoconstriction (Wedgwood et al., 2019).

Kikuchi et al. (2018) recently demonstrated that Selenoprotein P (SeP), an extracellular protein responsible for maintaining cellular metabolism, exhibits a 32-fold increase in human PAH-PASMCs compared with control PASMCs. They reported that SeP promoted PASMC proliferation and resistance to apoptosis through increased oxidative stress and mitochondrial dysfunction associated with dysregulated glutathione metabolism. In addition, SeP-knockout mice (SeP^–/–^) exposed to chronic hypoxia demonstrated significantly reduced PH and pulmonary vascular remodeling (Kikuchi et al., 2018).

Krüppel-like factor 4 (KLF4) plays an important role in the protection of endothelial cells (ECs) by regulating vasodilation, inflammation, coagulation, and oxidative stress. In a recent study, Ban et al. (2019) demonstrated that KLF4 undergoes S-nitrosation in response to nitrosative stress in the human umbilical vein ECs, which is mediated by endothelin-1 and is inhibited by endothelin receptor antagonist Bosentan. Furthermore, they demonstrated that S-nitrosated KLF4 was significantly increased in lung tissues, along with decreased nuclear localization of KLF4 in rats with hypoxia-induced PH (Ban et al., 2019). In another study, Masri et al. (2008) hypothesized that ROS consumption of NO may contribute to low NO levels and development of PH in human lungs. They found that antioxidants, glutathione peroxidase and SOD activities were decreased in IPAH lungs compared to controls, while catalase and glutathione activities were similar among the groups (Masri et al., 2008).

In summary, there is growing evidence implicating oxidative stress in the pathogenesis of PH in pre-clinical and clinical studies. It is important to identify the expression and function of oxidative stress markers in specific cell types in the lungs and pulmonary vasculature in order to help devise novel targeted therapies.

## Contribution of Oxidative Stress to Rv Remodeling in Ph

Oxidative stress plays a key role in pulmonary vascular remodeling, which in turn increases the RV after-load leading to RV hypertrophy and eventual RV failure (Bello-Klein et al., 2018). Additionally, it has been shown that the RV is more vulnerable to oxidative stress than the LV (Reddy and Bernstein, 2015), potentially because the RV cannot upregulate manganese superoxide dismutase expression, an enzyme that combats ROS (Shults et al., 2018).

Formation of ROS in many animal models of PH are derived from NADP oxidase activity, xanthine oxidase, and endothelial NOS. As PH progresses, circulating monocytes accumulate in the pulmonary arterioles and start to generate ROS to induce cell proliferation and fibrosis in the RV and small pulmonary arteries (Demarco et al., 2010). In an experimental model of PH induced by MCT in rats, high fat diet resulted in high levels of free radical formation in the dilated RV (Khoo et al., 2012). In Sugen-hypoxia model of PH in rats, a significant increase in oxidative stress caused cardiomyocyte deterioration. Furthermore, oxidative stress promoted perivascular fibrosis, leading to RV remodeling (Woo et al., 2017). In a rat model of angio-proliferative PH, although RV pressures were similar between males and females, males had worse RV hypertrophy, fibrosis, dysfunction, and survival compared to females. The reduced RV fibrosis in females was likely due to protection against oxidative stress as it correlated with increased caveolin-1 and decreased endothelial nitric oxide (NO) synthase-derived superoxide (Rafikova et al., 2015).

A decrease in SOD3 causes an imbalance between oxidants and antioxidants, and may therefore lead to vascular remodeling and PH. In SOD3 knockout mouse model with silica-induced PH, RV pressures were significantly higher compared to the wild type control mice (Zelko et al., 2018). The isoform SOD2 stabilizes the superoxide anion to hydrogen peroxide, which is less likely to damage the RV vasculature (Maron and Abman, 2017). Hence, the commonly used animal models of PH demonstrate association of oxidative stress markers with the changes accompanying RV remodeling.

## Molecular Pathways Associated With Rv Oxidative Stress in Pulmonary Hypertension

There are several proposed pathways leading to RV oxidative stress in PH. PH creates ROS, which in turn causes carbonylation of annexin A1 protein that results in its degradation by proteasomes in the RV. Annexin A1 is an inhibitor of CBF/NF-Y, a transcription factor that functions to activate the GATA4 gene. The GATA4 gene is a regulator of hypertrophy in the RV. In animal models of PH, increased GATA4 expression in the RV has been observed (Park et al., 2010; Suzuki and Shults, 2017). In an RV transcriptomic analysis of MCT-induced PH in Sprague-Dawley rats, Annexin A1 was identified to be dysregulated. Furthermore, the transcriptomic analysis of the RV showed that CYP2El, a gene that combats oxidative stress, is downregulated in PH (Potus et al., 2018).

When the RV experiences pressure overload, it uses NO to decrease the afterload by promoting pulmonary vasodilation. NO synthase 2 (NOS2) is induced in cardiac fibroblasts under RV pressure overload. Induction of NOS2 has been associated with collagen formation, which causes fibrosis in the RV and impaired function. Oxidative stress and cytokines which are elevated in PH, result in increased NF-κB expression. The NOS2 promotor has a NF-κB binding site that promotes transcription of NOS2. This creates oxidative stress, ROS formation, and eventual collagen deposition, which leads to fibrotic formation in the RV (Boehm et al., 2018).

Galectin-3 (Gal-3), a biomarker for LV remodeling, has been shown to interact with NOX4-derived oxidative stress promoting cardiac fibrosis in the RV in PH patients (He et al., 2017). Budas et al. (2018) studied the effects of inhibition of redox-sensitive apical MAPK and apoptosis signal-regulating kinase 1 (ASK1) on RV remodeling in rats with PH. Inhibiting ASK1 reduced remodeling of the pulmonary vasculature and the RV. ASK1 inhibitor, GS-444217 was shown to improve cardiac function and reduce fibrosis. GS-444217 decreased phosphorylation of p38 and JNK (c-Jun N-terminal kinase), and reduced cardiac fibroblast activation. Fibrosis formation causes a further increase in the severity of symptoms in PH patients. In a study by Wang X. et al. (2017) rats with Sugen-induced PH developed RV hypertrophy with increased levels of oxidized glutathiones, xanthine, and uric acid in the RV, which suggested higher production of ROS by xanthine oxidase. Also, there was a 30-fold lower level of antioxidant α-tocopherol nicotinate in the RV (Wang X. et al., 2017).

Glucose-6-phosphate (G6P) dehydrogenase is a major source of NADP which is a substrate for NADP oxidase in the RV. If G6P is inhibited, it reduces hypertrophy in the RV. One possible inhibitor of G6P is a 17-ketosteroid called Dehydroepiandrosterone, which was also shown to improve LV diastolic function (Rawat et al., 2014).

Oxidative stress generates nitrotyrosine residues in tissue inhibitor of metalloproteinase (TIMPs) which frees active martix metalloproteinase (MMP). It was hypothesized that the imbalance in MMP to TIMP causes fibrosis and ultimately RVF. It was found that folic acid did increase TIMP-4 and decreased different isoforms of MMPs. In pulmonary arterial constriction models, this was the opposite where TIMP-4 was decreased and MMP was increased. As a result, folic acid decreased levels of ROS in the RV wall, interstitial fibrosis, and RV pressures affirming the correlation between the oxidative stress and the RVF through the MMP to TIMP ratio (Qipshidze et al., 2012).

Ikeda et al. (2014) measured expression levels of oxidative stress related genes in the RV 24 h after pulmonary arterial constriction. Oxidative stress genes, such as p47phox and NOX2, were upregulated in the RV. Rho kinase (ROCK2) was rapidly induced after pulmonary arterial constriction in the RV free wall. In dominant negative Rho kinase mice, there was no presence of oxidative species. The timing between these steps are significant because it illustrates a correlation between Rho kinase, high pressures in the RV, and oxidative species (Ikeda et al., 2014). A possible treatment for Rho kinase overexpression is dehydroepiandrosterone (DHEA), a steroid, which has been shown to decrease NADPH levels in the RV. DHEA reduced the activity of Rho kinase, which in turn resulted in reduction of active forms of STAT3 and NFATc3 leading to rescue of RV remodeling. Overall the antioxidant activity of DHEA preserved contractility and slightly reduced high RVSP resulting from PH (Alzoubi et al., 2013).

A study by Dunlop et al. (2014) previously found that extended exposure to inhaled NO and chronic hypoxia causes a complete RV systolic dysfunction. A follow up study was done using therapeutic hypercapnia (10% CO2) in order to block the Interleukin (IL)-1α pathway which in turn inhibits NOS-2. This causes a complete normalization of RV hypertrophy (Dunlop et al., 2014). Chovanec et al. (2009) theorized another mechanism of hypercapnia, in which the release of oxygen radicals and NO are directly inhibited while in a hypoxic environment. In summary, there are multiple pathways leading to generation of oxidative stress in the RV secondary to pressure overload (see Table 1 for summary of pathways).

**TABLE 1:** Mechanistic pathways for the regulatory oxidative markers found in the right ventricle (RV) of pulmonary hypertension (PH) models.

**Oxidative stress marker**	**PH induction method**	**Pathways in the right ventricle**	**Source**
↑Nit-Tyr, ↑MMP, and ↓TIMP-4	Pulmonary artery constriction	RV-pressure overload by pulmonary arterial constriction → Increased ROSs → Oxidative stress generates nitrotyrosine residues in TIMPs → Increase in MMP, decrease in TIMP-4 → Mitophagy → RVF	Qipshidze et al., 2012
↑GATA4	Chronic hypoxia	PH → Pressure overload → ROS → Carbonylation of Annexin A1 → Annexin A1 degraded by proteasome → Annexin A1 no longer inhibits CBF/NFY → GATA4 activated → RV hypertrophy	Park et al., 2010; Suzuki and Shults, 2017
↑p47phox, ↑NOX-2, and ↑ROCK	Pulmonary artery constriction	Pulmonary arterial constriction → Upregulation of p47phox, NOX-2, and ROCK → ROCK leads to RV dysfunction	Ikeda et al., 2014
↑Gal-3 and ↑NOX4	MCT	Gal-3, NOX4, and NOX4 derived oxidative stress significantly elevated in PH → Gal-3 interacts with NOX4 and NOX4 derived oxidative stress and mediates TGF-β1-induced cardiac fibrosis → RV remodeling	He et al., 2017
↑NOS2 and ↑NF-κβ	Pulmonary artery banding	Pressure overloaded RV → NF-κβ regulates NOS2 transcription → NOS2 induction → ROS formation → collagen deposition from fibroblasts → RV hypertrophy and dilation	Boehm et al., 2018
↑Ox Glut	Sugen5416 + ovalbumin immunization	↑Pulmonary arterial pressure from Sugen5416 injection and ovalbumin immunization → increase in RV oxidized glutathione, xanthine and uric acid → ROS production by xanthine oxidase → RV failure	Wang X. et al., 2017
↑Lip-Per	MCT	→Pulmonary arterial pressure from MCT → Increase in ROS → Lipid Peroxidation and RV hypertrophy	Puukila et al., 2017
↑NOX-1, ↑Ox- Glut, and ↑Lip-Per	MCT	MCT induced PH → Increase in NOX-1, Ox Glut, and Lip-Per → Increased diameter of RV with impaired contractile function	Türck et al., 2018

*Nit-Tyr, nitrotyrosine; MMP, matrix metalloproteinase; TIMP, tissue inhibitor of metalloproteinase; NOX, NADPH oxidase; ROCK, Rho kinase; NOS, nitric oxide synthase; Ox Glut, oxidized glutathiones; Lip-Per, lipid peroxidation.*

## Targeting Oxidative Stress in Rv Remodeling Associated With Pulmonary Hypertension

### Pharmacological Treatments

A number of therapeutic strategies have been tested to alleviate the effects of oxidative stress in PH. Antioxidants are the primary agents that inhibit oxidation of biomolecules by ROS. However, oxidative stress regulation in the RV can be controlled by epigenetics, and a diverse group of protein interactions. Therefore solely administering antioxidants in bulk amounts is unlikely to be effective (Suzuki and Shults, 2017).

Many new therapies have been tested that simultaneously ameliorate the effects of both oxidative stress and PH. Dihydroartemisinin (DHA), an agent that has anti-inflammatory, anti-malaria, and anti-tumor effects has been shown to reduce the symptoms of MCT-induced PH. DHA inhibits pulmonary arterial EC proliferation and reduces oxidative stress by increasing SOD expression and reducing ROS (Yu et al., 2018). PPARα agonist fenofibrate (FF) is another discovered therapy against oxidative stress and PH. In MCT-induced PH rats, FF was orally administered to the rats 3-days post MCT. FF reduced RV hypertrophy, oxidative stress, ROS, NADPH Oxidase (NOX-1) expression, and increased the Bcl2/Bax ratio in the RV that was caused by PH (Galhotra et al., 2018). Trapidil, a vasodilator, has also been shown to mitigate the effects of oxidative stress and PH. Increased lipid and glutathione peroxidation as well as an increase in RV diameter has been reported in MCT model of PH in Wistar rats. Trapidil reduced NOX-1 activity, reduced glutathione oxidation in the RV, and reduced the diameter of the RV (Türck et al., 2018). Rats treated with high doses of iron caused pathological remodeling in the RV. The oxidative stress that causes the hypertrophy was mediated by Angiotensin II Type 1 receptor (AT1). An AT1 antagonist was administered orally and was shown to restore vascular function (Bertoli et al., 2018). It has been shown that α1-adrenergic receptor (α1-AR) antagonist A61603 improves left ventricular failure. In the bleomycin model of RV failure, A61603 increased cellular SOD1 and decreased NOX4, myocyte necrosis, and fibrosis in the RV (Cowley et al., 2017). Pterostilbene (PTS), a phytophenol, paired with hydroxypropyl-β-cyclodextrin (HPβCD) at high doses completely prevents RV failure and hypertrophy caused by PH, while protecting systolic function by reducing NADPH oxidase-dependent superoxide anions (Dos Santos Lacerda et al., 2017).

According to a study by Zimmer et al. (2017) it was initially believed that aerobic exercise would increase RV function in MCT-induced PH rats. However, they found no change in oxidative stress due to stable levels of eNOS activity (Zimmer et al., 2017). Contrary to the findings from Zimmer et al. (2017) aerobic exercise has been shown to promote hydrogen peroxide and vascular endothelial growth factor which ultimately improve RV function, in spite of ROS created by hydrogen peroxide. However, it is not specified if other antioxidant markers are associated with aerobic exercise (Colombo et al., 2016). In addition, aerobic exercise at early stages of PH decreases mitochondrial oxidative stress, demonstrating cardioprotective effects (Moreira-Gonçalves et al., 2015). Each study focused on a different oxidative stress marker, which led them to different conclusions on the effect of exercise on oxidative damage. However, all three studies have shown that RV hypertrophy is unaffected by aerobic exercise.

EUK-134, a known antioxidant, reduced cardiomyocyte hypertrophy and decreased end systolic volume in MCT-induced PH rats (Redout et al., 2010). Thus, this was one of the first studies that demonstrated the importance of ROS in contractile dysfunction due to PH and how antioxidants such as EUK-134 can be used for PH treatment.

### Herbal Treatments

Many of the novel therapies used to target oxidative stress and PH simultaneously, are derived from natural herbs and plants. One such therapy that showed similar effects to FF is ocimum sanctum (OS), a plant predominantly found in India. Similar to FF treatment, OS has shown a decrease in both RV hypertrophy and NOX-1 expression and an increase in Bcl2/Bax ratio in the RV tissue (Meghwani et al., 2018). The stem bark of a plant called Terminalia arjuna and Carvacrol, an oil in oregano and thyme, was also used to increase the Bcl2/Bax ratio in the pulmonary artery SMC (Zhang et al., 2016; Meghwani et al., 2017). Similar to the previously mentioned therapies, Copaiba oil has also been shown to decrease RV hypertrophy, and reduce oxidation of proteins and lipids in the RV of the MCT group (Campos-Carraro et al., 2018). In hypoxia-induced PH rats, Trimethoxystilbene (a novel polypehnol found in grapes and red wine with known anti-inflammatory effects) treatment prevented RVH by inhibiting the NOX/VPO1 pathway that mediates oxidative stress (Liu et al., 2014). NOX and VPO1 work together to cause higher levels of peroxide, which leads to smooth muscle cell proliferation (Brandes, 2011).

Grape seed procyanidin extract (GSPE) has been shown to prevent hypoxia-induced PH through antioxidant properties resulting in decreased RVSP and RV dilation. GSPE increased SOD and decreased NOX4 mRNA levels simultaneously, which decreased ROS production in pulmonary arterial SMC (Jin et al., 2016). Sesamin, an extract from sesame seeds, was used to inhibit NOX2 and NOX4 in the pulmonary vessels. This consequentially lowered RVSP and RV hypertrophy index (Li et al., 2015). The decrease of NOX4 and increase of SOD1 balances the antioxidant imbalance caused by PH. Secoisolariciresinol diglucoside (SDG), derived from flaxseed, was given to MCT rats with RV hypertrophy and increased lipid peroxidation. It did not decrease RV hypertrophy, however, it decreased ROS levels and SOD activity in the RV. When rats were pretreated with SDG, it was more effective in decreasing RV dysfunction (Puukila et al., 2017). When MCT rats were treated with Withania somnifera, a clinical antioxidant, oxidative markers such as levels of ROS were decreased in the lungs, which led to decreased RVSP (Kaur et al., 2015). The therapeutic effects of protandim, which enables Nrf2 to upregulate the expression of genes that code for antioxidants, were examined in Su/Hx model in rats. Protandim was found only to protect against RV failure, without affecting other PH symptoms like angio-obliteration caused by PH (Voelkel et al., 2013). This is important because a well-functioning RV is an important prognostic indicator of survival in PH patients.

In summary, there is a growing body of evidence demonstrating the association of oxidative stress to the development of RV remodeling in PH. Investigators have tested novel therapies targeting oxidative stress markers and molecular pathways to treat experimental RV failure. Further pre-clinical and clinical research is needed to validate the efficacy of these experimental therapies for targeting RV oxidative stress in PH.

## Past Failures of Antioxidant Ph Therapies in the Rv and Lungs

A variety of antioxidant therapies have been tested for the treatment of pre-clinical PH. Some studies have focused on targeting oxidative stress in the lungs, whereas others have focused more on the effects of antioxidant therapies in the RV. RV failure is the primary cause of death in PH patients, and although treatment of patients with vasodilator antioxidants may reduce pulmonary vascular resistance, this effect may not be accompanied by improvements in the RV (Drake et al., 2013).

Antioxidant therapies targeting the lungs and RV in PH have shown prior failures. NO can modulate cellular and physiological processes to limit oxidative stress. Saugstad (2000) argued that NO can act as an antioxidant and pro-oxidant depending on the dose of NO administered and the presence of other oxidants. Previous rat studies found that NO in high concentrations induces free radical mediated injury in rat lungs. Oxidative stress was only partially treated in the hyperoxic condition, which in itself, helped exacerbate the disease (Saugstad, 2000). L-arginine, a substrate needed to produce endogenous NO was studied to see whether it prevents RV and pulmonary vascular hypertrophy in hypoxic rats. Long term oral L-arginine failed to prevent RV and pulmonary vascular hypertrophy in the rats with hypoxia-induced PH (Laursen et al., 2008). Horstman et al. (1998) used a direct administration of low concentrations of inhaled NO and found that inhaled NO attenuates RV hypertrophy and vascular remodeling secondary to hypoxia, but has no such effects in MCT-induced PH. This shows that the effectiveness of inhaled NO in attenuating remodeling in PH is dependent on the mechanism by which PH was induced. Another group also found mechanism-dependent results when they looked to make a determination between vasodilator doses of inhaled NO and the prevention of progression of PH in MCT rats. In MCT-induced PH, there was a decrease in pulmonary artery pressure. However, in chronic hypoxic conditions inhaled NO did not prevent development of PH with no changes in the RV (Maruyama et al., 1997).

The use of continuous inhalation of NO as a treatment was also used in a study of rats exposed to chronic hypoxia followed by normoxia, but there was no effect on alleviating pulmonary vascular remodeling in the PH lungs (Jiang et al., 2004). Prior studies on the umbilical vein endothelial cells led to the belief that NO can decrease malignant ET-1 overexpression which in theory should minimize the effects of oxidative stress. However, in a group of rats with hypoxia induced PH, investigators found that NO had no impact on the increased ET-1 expression in rat lungs with PH induced by hypoxic conditions (Earley et al., 2002).

A novel attempt at using the administration of chronic oxygen, instead of NO, was used as a possible antioxidant therapy for PH. However, results showed that the high influx of oxygen resulted in increased RV inflammation, pulmonary arterial hypertrophy, and SOD activity. Rats that received MCT and oxygen therapy recruited leukocytes that promoted RV inflammation and failure. And despite increases in SOD activity, antioxidants were not able to sufficiently counteract the oxidative stress caused by the combination of MCT and chronic oxygen therapy (Fujita et al., 2018).

As discussed earlier in this review, protandim which upregulates the expression of genes encoding antioxidants such as SOD and heme oxygenase-1 (HO-1), was found only to protect against RV failure, without affecting pulmonary vascular angioobliterative lesions caused by PH. The authors argued that antioxidants may affect pulmonary vascular remodeling when administered early during the development of the pulmonary vascular lesions while antioxidants may impact RV failure even late in the disease process (Voelkel et al., 2013).

Several studies have demonstrated the efficacy of statins in experimental models of PH, by reducing oxidative stress and inflammation, inhibiting PASMC proliferation, and inducing PASMC/PAEC apoptosis and vasodilatation (Nishimura et al., 2002, 2003; Taraseviciene-Stewart et al., 2006; Girgis et al., 2007). These results were also supported by observational studies in PH patients (Kao, 2005; King and Day, 2011). However, a phase-II randomized clinical trial of aspirin and simvastatin for PAH (ASA-STAT) failed to demonstrate a significant effect on the six-minute walk distance between simvastatin and placebo groups (Kawut et al., 2011).

To summarize, although antioxidants have shown promise in the treatment of experimental PH and RV failure, there have been reports of failure of some of these therapies. Understanding the precise role of oxidative stress in the pathogenesis and progression of PH and RV failure will help in developing targeted and effective antioxidant therapies.

## Conclusion

Long standing PH leads to RV hypertrophy and RV failure. Accumulating evidence suggests involvement of oxidative stress in the development and progression of RV remodeling associated with PH. Targeting RV oxidative stress associated with PH may lead to the development of novel therapies for RV failure secondary to PH.

## Author Contributions

MM, CM, AW, TL, and ME contributed in drafting and revising the manuscript. SU contributed in drafting and revising the manuscript, and provided the supervision.

## Conflict of Interest

The authors declare that the research was conducted in the absence of any commercial or financial relationships that could be construed as a potential conflict of interest.
